# PlantID – DNA-based identification of multiple medicinal plants in complex mixtures

**DOI:** 10.1186/1749-8546-7-18

**Published:** 2012-07-28

**Authors:** Caroline Howard, Eleni Socratous, Sarah Williams, Eleanor Graham, Mark R Fowler, Nigel W Scott, Paul D Bremner, Adrian Slater

**Affiliations:** 1 Biomolecular Technology Group, Faculty of Health and Life Sciences, De Montfort University, Leicester, LE1 9BH, UK; 2 East Midlands Forensic Pathology Unit, University of Leicester, Leicester Royal Infirmary, Leicester, LE2 7LX, UK; 3 Department of Chemical and Forensic Science, School of Life Sciences, Northumbria University, Newcastle upon Tyne, NE1 8ST, UK

## Abstract

**Background:**

An efficient method for the identification of medicinal plant products is now a priority as the global demand increases. This study aims to develop a DNA-based method for the identification and authentication of plant species that can be implemented in the industry to aid compliance with regulations, based upon the economically important *Hypericum perforatum* L. (St John’s Wort or *Guan ye Lian Qiao*).

**Methods:**

The ITS regions of several *Hypericum* species were analysed to identify the most divergent regions and PCR primers were designed to anneal specifically to these regions in the different *Hypericum* species. Candidate primers were selected such that the amplicon produced by each species-specific reaction differed in size. The use of fluorescently labelled primers enabled these products to be resolved by capillary electrophoresis.

**Results:**

Four closely related *Hypericum* species were detected simultaneously and independently in one reaction. Each species could be identified individually and in any combination. The introduction of three more closely related species to the test had no effect on the results. Highly processed commercial plant material was identified, despite the potential complications of DNA degradation in such samples.

**Conclusion:**

This technique can detect the presence of an expected plant material and adulterant materials in one reaction. The method could be simply applied to other medicinal plants and their problem adulterants.

## Background

The quality of Chinese medicines (CM) has been questioned because of “continuing evidence of an international trade in herbal remedies made to an unreliable standard” [1-3], and there is increasing international demand for regulation of phytomedicines and definitive quality standards [4,5]. In the European Union (EU), the Traditional Herbal Medicines Directive (Directive 2004/24/EC) regulates medicinal plant products for human use, and all medicinal plant products must now hold a Traditional Herbal Registration (THR) certified by a logo on all packaging. To gain a THR, many factors must be certified, such as the identification and authentication of the medicinal plant material upstream of manufacturing and processing. This is currently achieved by morphological and chemical methods, both of which are time-consuming and cost-intensive [6].

DNA-based methods are a preferred alternative, because they are more efficient, less expensive and time-consuming, require less plant material, and can reliably distinguish materials to the species level [6-9]. However, the current identification methods (DNA-based and chemical) are not capable of resolving mixtures of several plant species simultaneously.

Identification of species independently and concurrently in complex mixtures has become increasingly important for regulators based on two main factors. First, adulteration and contamination occur, particularly with rare and expensive medicinal plant species that are substituted with less valuable alternatives [10,11]. These alternatives may have no biological activity or might have detrimental health implications. Therefore, the target plant and the dangerous adulterant must be identified to assess the safety of products. Second, synergistic polyherbal formulations are fundamental to the practices of CM, in that the use of specific combinations of medicinal plant materials results in an enhanced outcome, *i.e.* the whole being greater than the sum of its parts. Each of the plants included must be identified to authenticate such preparations.

As a pilot study for the design of industry-standard DNA-based identification assays, *Hypericum perforatum* L. (St John’s Wort or *Guan ye Lian Qiao*), which is used for its anti-inflammatory and antimicrobial properties in CM and for the treatment of mild to moderate depression in Europe [12], was selected as the target for the design of a new assay.

The nuclear ribosomal internal transcribed spacer (nrITS) regions were used as targets for the design, and the sequences from 15 *Hypericum* species were aligned and analysed to identify the most divergent regions. PCR primers were then designed to anneal specifically to these regions of the different species. Candidate primers were selected such that the species-specific product of each PCR differed in size and could be resolved by capillary electrophoresis with the fluorescently labelled primers.

This method is capable of detecting the closely related species *Hypericum androsaemum*, *Hypericum athoum*, *Hypericum ascyron* and *Hypericum perforatum* individually and in any combination, from within a mixture of DNA from seven *Hypericum* species. This technique has the power to both confirm the presence of the expected plant material and detect the adulterant material in one reaction. The method of design can be replicated for any other medicinal plants and their problem adulterants.

This study aims to develop a DNA-based method for the identification and authentication of plant species that can be implemented in the industry to aid compliance with regulations, through the discrimination of several different *Hypericum* species, based on a similar design to that used by Tobe and Linacre [13] for identifying mammalian species. These species represent a worst-case scenario for discrimination, as they are extremely closely related.

## Methods

### Primer design

Species-specific primers were designed using Allele ID software (PremierBiosoft, USA) [14] available at http://www.premierbiosoft.com/bacterial-identification/index.html. The *Hypericum* nrITS sequences used for the design template were obtained from GenBank at http://www.ncbi.nlm.nih.gov/, with accession numbers and species as follows: AJ414728, *Hypericum calycinum*; AY555839; *Hypericum perforatum* subsp. *perforatum*; AY555842. *Hypericum maculatum*; AY555846, *Hypericum athoum*; AY555849, *Hypericum ascyron*; AY555853, *Hypericum kouytchense*; and AY573012, *Hypericum androsaemum*. Potential interactions between primers were assessed using AutoDimer v.1 Software (National Institute of Standards and Technology, USA) [15] available at http://yellow.nist.gov:8444/dnaAnalysis/primerToolsPage.do.

### Initial PCR

The DNA samples used as templates for the initial primer testing were supplied by the Royal Botanic Gardens at Kew (UK) (Table1). The nrITS1 region was amplified using primers ITS1 (5′-TCCGTAGGTGAACCTGCGG-3′) and ITS4 (5′-TCCTCCGCTTATTGATATGC-3′) [16]. The PCRs were conducted with the GeneAmp® High Fidelity PCR System (Applied Biosystems, USA), using a final volume of 50 μL. The PCRs in 0.2-mL polypropylene tubes consisted of GeneAmp High Fidelity PCR Buffer (without MgCl_2_) (1×), MgCl_2_ (2.5 mM), GeneAmp High Fidelity Enzyme Mix (2.5 U), primers (0.1 μM each), dNTPs (0.1 μM each), nuclease-free water and template DNA (0.7–1 μg). A GeneAmp PCR System 9700 thermal cycler (Applied Biosystems, USA) was used with the following programme: initial denaturation step of 7 min at 95°C; 30 cycles of 1 min at 95°C, 30 s at 60°C and 1 min at 72°C; and final extension period of 7 min at 72°C.

**Table 1:** Voucher numbers and species for samples from the DNA Databank of the Royal Botanic Gardens at Kew

**Kew DNA Bank Voucher number**	**Hypericum species**	**Authority**
13854	*H. androsaemum*	L.
13866	*H. kouytchense*	H.Lév.
13896	*H. maculatum*	Crantz
13921	*H. perforatum*	L.
13923	*H. athoum*	Boiss.&Orph.
13929	*H. calycinum*	L.
13993	*H. ascyron*	L.

### Primer validation

The initial testing of the species-specific primers was conducted by conventional PCR using the High-Fidelity PCR amplicons as DNA templates. The products from these amplifications were diluted to a suitable working concentration (*H. athoum*, 1 × 10^-4^ dilution; remainder, 1 × 10^-5^ dilution). This enabled all possible combinations to be tested against vouchered DNA samples, which were in limited supply. To test cross-amplification, non-target DNA panels were created with the remaining six *Hypericum* species (Table2). This meant that six species could be eliminated for cross-amplification in one reaction. As each species represented one-sixth of the DNA present in the panel (*e.g.* 5ng each, totalling 30 ng), the DNA used to check for amplification of the target DNA was diluted to produce the same final concentration (*e.g.* 5 ng).

**Table 2:** Construction of non-target DNA panels from initial High-Fidelity PCR amplifications of vouchered DNA samples

**Panel**	***H. androsaemum***	***H. kouytchense***	***H. maculatum***	***H. athoum***	***H. calycinum***	***H. ascyron***	***H. perforatum***
Non-and	x	✓	✓	✓	✓	✓	✓
Non-ath	✓	✓	✓	x	✓	✓	✓
Non-asc	✓	✓	✓	✓	✓	x	✓
Non-perf	✓	✓	✓	✓	✓	✓	x
Multiplex	✓	✓	✓	✓	✓	✓	✓

The initial PCR products were diluted by 1 × 10^-5^ (except *H. athoum*, 1 × 10^-4^) and equal volumes of each sample were added to the panel mixtures, as indicated by a tick. The panels are described by the name of the species that is not present, indicated by a cross. These panels were then used to test the species-specific primers for each target for cross-amplification, allowing six DNA samples to be tested simultaneously. The panel used to test the multiplex reaction contained all the available samples, and is named Multiplex.

The reactions consisted of Green GoTaq® Flexi Buffer (Promega, USA) (1×), MgCl_2_ (2.5 mM), GoTaq® DNA Polymerase (Promega, USA) (1.25 U), relevant primers (0.1 μM each), dNTPs (0.1 μM each) and template DNA (1 μL of appropriate sample dilution) made up to a final volume of 25 μL with nuclease-free water in 0.2-mL polypropylene tubes. The GeneAmp PCR System 9700 thermal cycler was used with the following programme: initial denaturation step of 7 min at 95°C; 30 cycles of 1 min at 95°C, 30 s at 60°C and 1 min at 72°C; and final extension period of 7 min at 72°C. Combinations requiring further optimisation were run on a gradient of annealing temperatures from 55°C to 69°C, with all other parameters as described above. Reactions without template DNA were utilised as controls. The PCR products were electrophoresed in 50-mL 3% (w/v) agarose, 0.5× TBE gels with 1 μL of SYBRsafe™ (Invitrogen, USA) DNA stain at 90 V for ~30 min and analysed using an Illuminator with a ChemiDoc XRS Camera and Quantity One software (Bio-Rad, USA).

### Commercial samples

The commercial materials were capsules filled with dried, ground plant material from three companies, which were labelled as containing the following: Company A, 334 mg St John’s Wort extract per capsule; Company B, 300 mg of St John’s Wort pure powdered herb per capsule; and Company C, 333 mg of St John’s Wort (*H. perforatum*) standardised herb extract and 114 mg of other plant extracts and concentrates.

### DNA extraction

DNA extraction was carried out utilising a DNeasy Plant Mini Kit (Qiagen, USA) and a TissueLyser (Qiagen, USA). Manufacturer’s instructions were followed with the exception of conducting two disruption steps of 1 min at 30 Hz after the addition of 400 μL of Buffer AP1 and 4 μL of RNase A to the sample at the beginning of the procedure. Dried material (0.02 g) from within the capsules was used and the resultant DNA samples stored at 2-5°C.

### Multiplex PCR

A DNA panel was created that included all seven *Hypericum* species available to optimise the multiplex PCR (Table2). Multiplex PCRs with a final volume of 10 μL were conducted in 0.2-mL polypropylene tubes using a Multiplex PCR Kit (Qiagen Inc., USA). The reactions consisted of Multiplex PCR Master Mix (Qiagen Inc., USA) (1×), primer mix (*H. perforatum*, 500 nM; *H. athoum*, 110 nM; *H. androsaemum*, 80 nM; *H. ascyron*, 400 nM) (IDT, USA), template PCR product (0.4 μL at the working concentration) and nuclease-free water. The forward primers were labelled with 6-carboxyfluorescein (FAM). Reactions without either template PCR product or primers were used as controls. The PCR cycling parameters were: initial denaturation step of 15 min at 95°C; 30 cycles of 30 s at 94°C, 90 s at 64°C and 60 s at 72°C; and final extension period of 30 min at 60°C. Genomic multiplex reactions were conducted with a mixture containing equal concentrations of genomic DNA from *H. androsaemum*, *H. athoum*, *H. perforatum* and *H. ascyron* (Table1).

### Capillary electrophoresis

The fluorescently labelled multiplex PCR products were analysed in an ABI Prism™ 3130 Genetic Analyzer (Applied Biosystems, USA) using a 30-cm capillary and Performance Optimised Polymer 4 (Applied Biosystems, USA). The run module used consisted of a 12-s injection at 1.2 kV, followed by electrophoresis at 60°C and 15 kV for 25 min. Each multiplex PCR product (1 μL) was diluted with 8.5 μL of Hi Di™ Formamide and 0.5 μL of GeneScan™-500 ROX™ size standard (Applied Biosystems, USA) before the capillary electrophoresis. GeneMapper® ID v3.2 fragment analysis software (Applied Biosystems, USA) was used.

**Figure 1:**
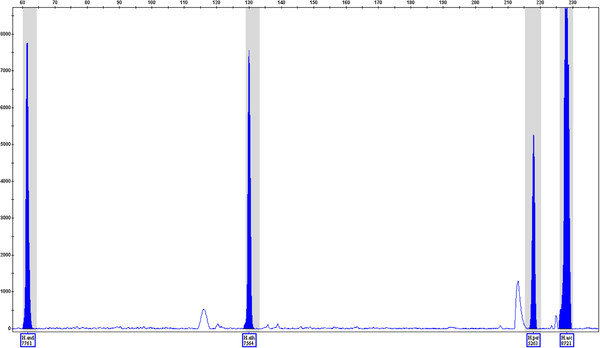
**ITS amplification products from all seven*Hypericum*samples were included as templates in a multiplex PCR.** The multiplex panel composition is described in Table2. The species-specific products from all of the four target species are shown (*H. androsaemum*, 67 bp; *H. athoum*, 131 bp; *H. perforatum*, 222 bp; and *H. ascyron*, 231 bp), each being automatically identified using the GeneMapper software.

The candidate primers were introduced to the multiplex reaction. This was carried out with the mixture of all seven *Hypericum* species nrITS sequences (Table2), and the products were separated by capillary electrophoresis (Figure1). The concentration of each primer pair was optimised to account for the efficiency differences found when introducing the primers into a multiplex reaction. Panels and bins were created in GeneMapper ID v.3.2 (Applied Biosystems, USA) following the instructions in the software, to allow for automatic recognition and labelling of amplicons falling within ± 0.5 bp of the determined fragment size for each species of interest.

## Results

**Figure 2:**
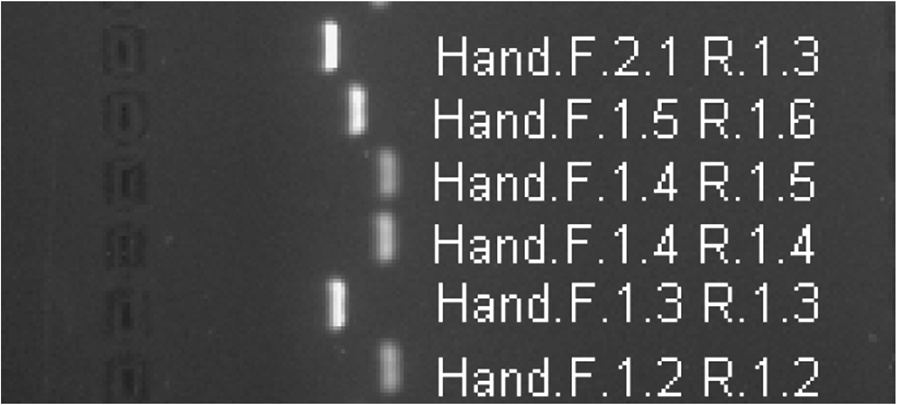
**Image of gel containing the PCR products from the*H. androsaemum*primers (as labelled) and*H. androsaemum*template DNA.** The product sizes from top to bottom are 137, 98, 63, 67, 135 and 65 bp. The primer pair Hand.F.1.4 and R.1.4 was selected for the PlantID assay.

The nrITS sequences from seven *Hypericum* species were aligned and analysed to provide the basis for the species-specific primer design. PCR primers were designed for regions where the sequences of the four target species (*H. androsaemum*, *H. ascyron*, *H. athoum* and *H. perforatum*) differed from all the other species. The sequences of each primer pair were species-specific, because they matched only one sequence from all the input sequences. A total of 19 primer pairs were designed, all of which produced amplicons of the expected size when tested with the target DNA template in conventional PCR: six pairs for *H. androsaemum*; four pairs for *H. ascyron*; four pairs for *H. athoum*; and five pairs for *H. perforatum* (Table3). The results for the six *H. androsaemum* pairs are shown in Figure2.

**Table 3:** Candidate primer pairs for species-specific identification of the target DNAs

**Forward primer name**				**Sequence 5′ to 3′**	***Tm***	**Reverse primer name**				**Sequence 5′ to 3′**	***Tm***	**Amplicon length (bp)**
Hand	F	1	2	ACATCGTCGCCCCAAACC	67.0	Hand	R	1	2	CCATTATCCGCCCCATCCTC	66.5	65
Hand	F	1	3	AAATGTGATACTTGGTGTGAATTGC	64.7	Hand	R	1	3	CGAGGTGTTGGGTTTGGG	64.8	135
**Hand**	**F**	**1**	**4**	**CACATCGTCGCCCCAAAC**	**65.6**	**Hand**	**R**	**1**	**4**	**ACCATTATCCGCCCCATCC**	**65.9**	**67**
Hand	F	1	4	CACATCGTCGCCCCAAAC	65.6	Hand	R	1	5	TTATCCGCCCCATCCTCTTC	65.4	63
Hand	F	1	5	CGGCTGTCCTCCTGTTCATAAC	67.1	Hand	R	1	6	TCACACCAAGTATCACATTTCGCTAC	67.3	98
Hand	F	2	1	CGAAATGTGATACTTGGTGTGAATTG	65.1	Hand	R	1	3	CGAGGTGTTGGGTTTGGG	64.8	137
Hasc	F	1	2	TTCCTTCGGTTCATAACTAAAAC	60.9	Hasc	R	1	2	ACCCAATGAACTCGAAAGAG	61.7	225
**Hasc**	**F**	**1**	**4**	**GTGGCTTTCCTTCGGTTC**	**62.5**	**Hasc**	**R**	**1**	**2**	**ACCCAATGAACTCGAAAGAG**	**61.7**	**231**
Hasc	F	1	3	TTTCCTTCGGTTCATAACTAAAAC	61.5	Hasc	R	1	1	GAACTCGAAAGAGGCATTG	60.4	219
Hasc	F	1	5	TCCTTCGGTTCATAACTAAAAC	60.2	Hasc	R	1	1	GAACTCGAAAGAGGCATTG	60.4	217
Hath	F	1	1	CCCCGAAATTCCGATATCTC	61.8	Hath	R	1	1	CTTACAACCACCGCTAGTC	61.7	137
Hath	F	1	1	CCCCGAAATTCCGATATCTC	61.8	Hath	R	1	3	CAACCACCGCTAGTCGTG	64.6	133
Hath	F	1	1	CCCCGAAATTCCGATATCTC	61.8	Hath	R	1	4	CCGCTAGTCGTGGCTTTG	64.9	127
**Hath**	**F**	**1**	**3**	**GTGTCACACATCGTTGCC**	**63.2**	**Hath**	**R**	**1**	**3**	**CAACCACCGCTAGTCGTG**	**64.6**	**151**
Hper	F	1	1	TGTAACGCTCCCGGCTGTG	69.0	Hper	R	1	1	CCGATTGTCTCTTGCGAGATATC	65.0	273
**Hper**	**F**	**4**	**1**	**GGGGCTTCCTTCTGTTCATAAC**	**65.1**	**Hper**	**R**	**4**	**1**	**TCTTGCGAGATATCGGGATTTTG**	**64.9**	**222**
Hper	F	1	2	ATAAGAAGTGTAACGCTCCCGGCTGTG	72.5	Hper	R	1	1	CCGATTGTCTCTTGCGAGATATC	65.0	281
Hper	F	1	3	GAAGTGTAACGCTCCCGGCTGTG	71.9	Hper	R	1	1	CCGATTGTCTCTTGCGAGATATC	65.0	277
Hper	F	1	4	AGTGTAACGCTCCCGGCTGTG	71.2	Hper	R	1	1	CCGATTGTCTCTTGCGAGATATC	65.0	275

The selected pairs are indicated in bold. Candidate selection was based on specificity, and further analysis of theoretical interactions in the multiplex PCR and discrimination of amplicons by capillary electrophoresis were used to make the final selection.

**Figure 3:**
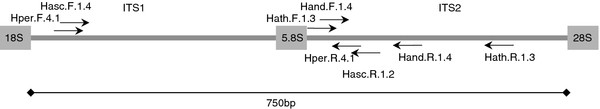
**Representation of the nuclear ribosomal coding region with the internal transcribed spacers, ITS1 and ITS2.** The relative annealing positions of the four selected primer pairs are shown.

The primer pairs were tested for cross-amplification against a panel of nrITS sequences from closely related *Hypericum* species, the non-target panel (Table2). In each case, panels were constructed using High-Fidelity PCR amplifications of the nrITS regions from vouchered species specimens. Primer pairs that gave a product with the nrITS of the target DNA, but not with the non-target DNA panel, were candidates for the multiplex reaction. Of the candidate primer pairs found for each of the four species, one primer pair per species was selected for the multiplex reaction (shown in Figure3, highlighted in Table3). These pairs were chosen based on analysis with the AutoDimer v.1 software, which indicated a low possibility of interactions between the candidate primers when all were introduced into a multiplex system. The candidate primers were also selected based on the size of the resultant amplicons, because the products must be sufficiently different in size to facilitate their separation by capillary electrophoresis. Consequently, a minimum length difference of 5 bp was considered.

**Figure 4:**
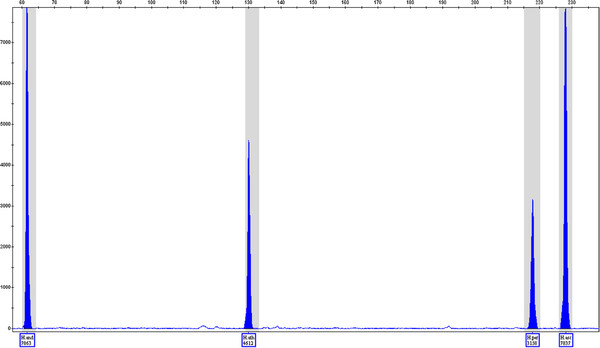
**Multiplex PCR products from a mixture of genomic DNA from the species*H. androsaemum*,*H. athoum*,*H. perforatum*and*H. ascyron*amplified with corresponding species-specific PCR primers.** Each peak is automatically identified and confirms the presence of the named DNA.

PCR products were used as the templates for the design and optimisation of the assay. But genomic DNA is likely to be the eventual target for the method. The assay was conducted with mixtures of genomic DNA from each of the target species to ensure that the assay was equally efficient for this type of sample (Figure4), producing a higher quality profile of peaks with fewer baseline anomalies.

The working assay was used to test DNA extracted from commercial preparations of St. John’s Wort sold in capsule or tablet form, produced by companies A, B and C. The three DNA samples were previously confirmed by conventional PCR of the ITS regions [17]. DNA degradation and/or shearing was observed in the samples from companies A and B, as the 750-bp ITS region could not be amplified, while a smaller amplicon (160 bp) within this region was amplified. This may have arisen because of the age and processed nature of the plant material. The sample from company C produced the full 750-bp ITS amplicon.

**Figure 5:**
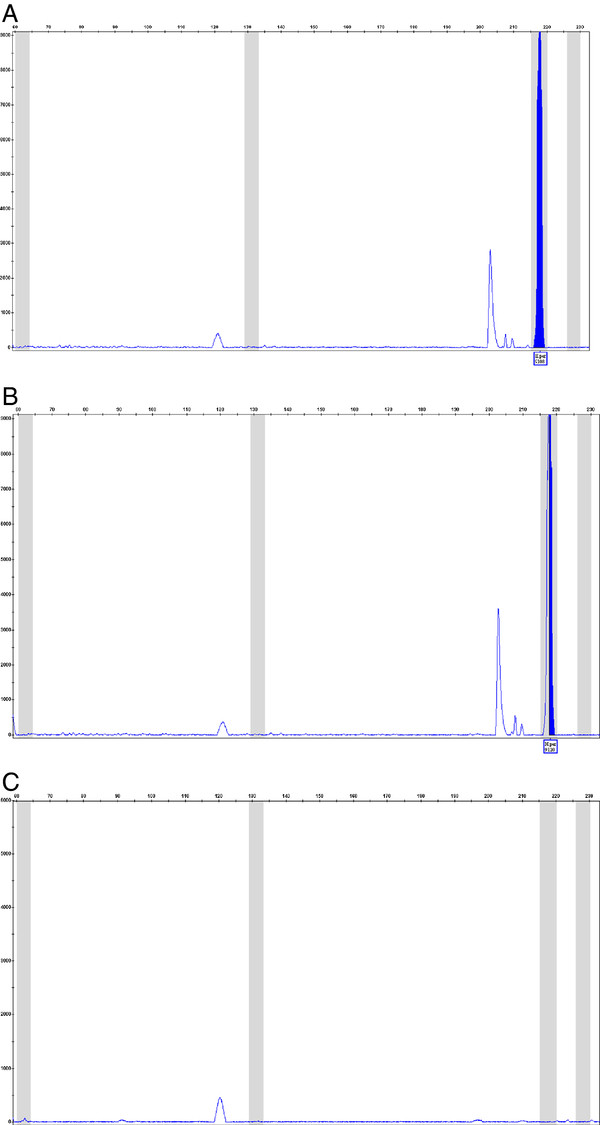
**Multiplex PCR product profiles from DNA extracted from three different companies products(companies A, B and C, panels labelled respectively).** The results show a positive peak for *H. perforatum* for companies **A** and **B**, but not for company **C.**

The multiplex reaction was carried out using DNA extracted from the three samples. The DNA extracted from the samples of companies A and B was identified as *H. perforatum*, whereas the DNA from the sample of company C was not identified as any of the *Hypericum* species in the assay (Figure5). The sample from company C is labelled as a mixture of plant extracts, and the amplified DNA may come from another species. However, *H. perforatum* was stated as the dominant plant component, and would have been expected to be the dominant DNA component.

## Discussion

The developed assay, PlantID, can detect four closely related species (*i.e.* the number of sequence differences between species within the nrITS region is minimal) simultaneously in one reaction within a mixture of seven species. Variation in the sequences used for the design confers the possibility for successful species-specific primer design, which is essential for this assay. The plant species in genuine cases of misidentification or adulteration may not be this closely related, and are likely to contain more DNA sequence variation.

During the design process, amplicons were ensured to be of the shortest possible length, thereby optimising the technique for use with degraded or fragmented samples following the miniSTR (Sequence Tagged Repeat) approach introduced by Butler in 2003 [18]. This enables the assay to be used with commercial products containing highly processed plant material. This is of particular importance when testing tablet forms of herbal medicines, as well as ingested samples collected during post-mortem [19].

The number of species identified using this technique could be dramatically increased by altering a few parameters. Although only one type of fluorescent label was used in this study, the system is capable of simultaneous detection of five fluorophores in a run. One of these fluorophores is reserved for the size standard, leaving four options for primer labelling, which can increase the detection yield by four-fold. In addition, the nrITS region alone was the basis for this “proof of concept” assay design. The use of more DNA regions would increase possibilities for unique annealing positions for primers, thereby increasing detection. The use of multiple DNA sequence regions for the assay design could also confer greater reliability. The technique could be further developed such that each species is identified by the presence of several peaks, which would greatly reduce the possibility of a false-positive result. The optimisation of this assay will aim to achieve the optimal multiplex reaction so that each peak produced is of equal intensity when the input DNA templates are at the same concentration. This would then produce a semiquantitative assay, with relative peak heights indicating which DNAs are present at the highest and lowest concentrations.

The evaluation of polyherbal preparations would benefit from the development of this type of assay, for which no other techniques can confirm the presence of each individual species. For example, Ayurvedic preparations, such as Dashmool, contain many different plant species that are highly processed, making them impossible to identify morphologically. Chemical analyses of such a mixed preparation containing many compounds from different species produce a highly complex profile. Substitution of the raw materials in this preparation is common and has led to the development of a DNA-based assay that can identify one species that should be in the preparation (*Desmodium gangeticum*) and two species that are often found as adulterants (*Desmodium velutinum* and *Desmodium triflorum*) [20]. Each of these is identified by an individual PCR, the product of which is then analysed by gel electrophoresis. However, the multiplex PlantID system could potentially identify all 10 different species that should be present in the preparation, and also test for species that are known to be used as adulterants, in one reaction.

## Conclusion

This technique can detect the presence of an expected plant material and adulterant materials in one reaction. The method could be simply applied to other medicinal plants and their problem adulterants.

## Abbreviations

EU, European union; ITS, internal transcribed spacer; STR, sequence tagged repeat; CM, Chinese medicine; THR, Traditional herbal registration.

## Competing interests

The authors declare that they have no competing interests.

## Authors’ contributions

CH conducted molecular procedures. ES conducted the capillary electrophoresis and analysed results relating to the design. SW conducted molecular work and performed optimisation. CH, EG, MF, NS, PB and AS designed the study and wrote the manuscript. All authors read and approved the final manuscript.
